# SARS-CoV-2 Entry Receptor ACE2 Is Expressed on Very Small CD45^−^ Precursors of Hematopoietic and Endothelial Cells and in Response to Virus Spike Protein Activates the Nlrp3 Inflammasome

**DOI:** 10.1007/s12015-020-10010-z

**Published:** 2020-07-20

**Authors:** Mariusz Z. Ratajczak, Kamila Bujko, Andrzej Ciechanowicz, Kasia Sielatycka, Monika Cymer, Wojciech Marlicz, Magda Kucia

**Affiliations:** 1 Stem Cell Institute at James Graham Brown Cancer Center, University of Louisville, 500 S. Floyd Street, Rm. 107, Louisville, KY 40202 USA; 2 Department of Regenerative Medicine, Center for Preclinical Research and Technology, Medical University of Warsaw, Warsaw, Poland; 3 Institute of Biology, Faculty of Exact and Natural Sciences, University of Szczecin, Szczecin, Poland; 4 Research and Developmental Center Sanprobi, Szczecin, Poland

**Keywords:** SARS-CoV-2, COVID19, Spike protein, ACE2, Nlrp3 inflammasome, VSELs, Hematopoietic stem cells, Cytokine storm, Pyroptosis

## Abstract

Angiotensin-converting enzyme 2 (ACE2) plays an important role as a member of the renin–angiotensin–aldosterone system (RAAS) in regulating the conversion of angiotensin II (Ang II) into angiotensin (1–7) (Ang [1–7]). But at the same time, while expressed on the surface of human cells, ACE2 is the entry receptor for SARS-CoV-2. Expression of this receptor has been described in several types of cells, including hematopoietic stem cells (HSCs) and endothelial progenitor cells (EPCs), which raises a concern that the virus may infect and damage the stem cell compartment. We demonstrate for the first time that ACE2 and the entry-facilitating transmembrane protease TMPRSS2 are expressed on very small CD133^+^CD34^+^Lin^−^CD45^−^ cells in human umbilical cord blood (UCB), which can be specified into functional HSCs and EPCs. The existence of these cells known as very small embryonic-like stem cells (VSELs) has been confirmed by several laboratories, and some of them may correspond to putative postnatal hemangioblasts. Moreover, we demonstrate for the first time that, in human VSELs and HSCs, the interaction of the ACE2 receptor with the SARS-CoV-2 spike protein activates the Nlrp3 inflammasome, which if hyperactivated may lead to cell death by pyroptosis. Based on this finding, there is a possibility that human VSELs residing in adult tissues could be damaged by SARS-CoV-2, with remote effects on tissue/organ regeneration. We also report that ACE2 is expressed on the surface of murine bone marrow-derived VSELs and HSCs, although it is known that murine cells are not infected by SARS-CoV-2. Finally, human and murine VSELs express several RAAS genes, which sheds new light on the role of these genes in the specification of early-development stem cells.

**Graphical Abstract:**
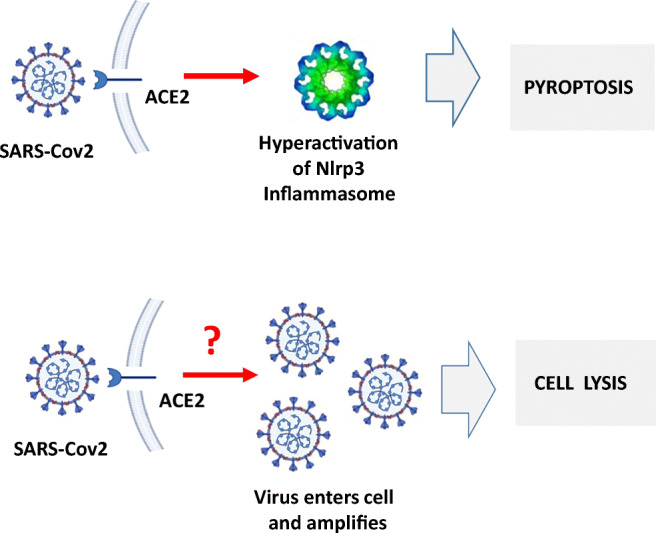
•Human VSELs and HSCs express ACE2 receptor for SARS-CoV2 entry. •Interaction of viral spike protein with ACE2 receptor may hyperactivate Nlrp3 inflammasome which induces cell death by pyroptosis. •SARS-CoV2 may also enter cells and eliminate them by cell lysis. •What is not shown since these cells express also Ang II receptor they may hyperactivate Nlrp3 inflammasome in response to Ang II which may induce pyroptosis. Our data indicates that Ang 1–7 may have a protective effect.

•Human VSELs and HSCs express ACE2 receptor for SARS-CoV2 entry.

•Interaction of viral spike protein with ACE2 receptor may hyperactivate Nlrp3 inflammasome which induces cell death by pyroptosis.

•SARS-CoV2 may also enter cells and eliminate them by cell lysis.

•What is not shown since these cells express also Ang II receptor they may hyperactivate Nlrp3 inflammasome in response to Ang II which may induce pyroptosis. Our data indicates that Ang 1–7 may have a protective effect.

## Introduction

The SARS-CoV-2 pandemic, with its high mortality, has become an urgent clinical problem. This infection damages several organs, including lungs, heart, blood vessels, kidneys, and intestines and may lead to a fatal complication known as a “cytokine storm”, which results in uncontrolled hyperactivation of the innate immunity-initiated response. SARS-CoV-2 may *i)* directly infect human cells and lead to their lysis or damage or *ii)* upregulate mediators of the renin–angiotensin–aldosterone system (RAAS), which may eliminate cells in a Nlrp3 inflammasome hyperactivation-mediated manner by pyroptosis [1–5].

It is well established that SARS-CoV-2 enters human cells after binding to the angiotensin-converting enzyme 2 (ACE2) receptor and utilizes a spike protein (S) for attachment and entry into the cells [4, 5]. The viral S protein must be primed by transmembrane protease 2 (TMPRSS2) to facilitate interaction with ACE2 and the subsequent fusion of viral and cellular membranes [8]. The ACE2 receptor has been found on the surface of many cells, and its physiological role is to processes conversion of angiotensin II (Ang II) to angiotensin (1–7) (Ang [1–7]) [1–3, 9]. These two members of the RAAS family have opposite biological effects on target cells and activate the angiotensin 1 receptor (AT_1_R) and MasR, respectively [10]. Activation of AT_1_R during SARS-CoV-2 infection has detrimental effects, inducing fibrosis, an increase in reactive oxygen species (ROS) release, vasoconstriction, and gut dysbiosis. By contrast, the effect of MasR activation is overall protective, ant-fibrotic, antioxidant, and vasodilatory.

It has already been demonstrated that hyperactivation of AT_1_R by Ang II may lead to excessive activation of the Nlrp3 inflammasome and cell death by pyroptosis in lung epithelium cells, endothelium, and cardiomyocytes [11–14]. By contrast, after binding to MasR, Ang (1–7) displays the opposite effect and has been demonstrated to stimulate proliferation of skeletal muscle and hematopoietic cells [6, 15]. Unfortunately, because of ACE2 internalization during SARS-CoV-2 infection Ang II is not processed to Ang (1–7).

The Nlrp3 inflammasome triggers an inflammatory immune response via intracellular caspase 1, which leads to *i)* release of the potent pro-inflammatory cytokines interleukin 1β (IL-1β) and interleukin 18 (IL-18) and *ii)* mediates the release of several biologically active danger-associated molecular pattern molecules (DAMPs) by creating gasdermin D (GSDMD) pore channels in cell membranes [16–18]. This initiates a sequence of events leading to amplification of the innate immune system response and activation of its major humoral arm, the complement cascade (ComC) [19, 20].

Based on the aforementioned, SARS-CoV-2 may enter and damage cells that express ACE2 entry receptor or damage them by hyper-activation of the Ang II–AT_1_R axis [21], which may lead to excessive Nlrp3 signaling and pyroptosis [22, 23]. Since many types of cells, including HSCs and EPCs, express both ACE2 and AT_1_R, this mechanism suggests that the stem cell compartment may be a direct target for damage by the virus. This also raises the question of potential long-term deleterious effects of infection on organ and tissue regeneration. To address this important question, we became interested in the potential effect of SARS-CoV-2 on the population of CD45^−^ cells, known as very small embryonic-like stem cells (VSELs), residing in postnatal tissues. These cells, which have been described and confirmed by several independent laboratories [24–35], may become specified into HSCs and EPCs and thus may revive the old concept of the presence of postnatal hemangioblasts in bone marrow [36–40]. Interestingly, several previous reports have indicated that hemangioblasts may be regulated by RAAS mediators [41].

Here we demonstrate for the first time that human VSELs express ACE2, the spike protein-priming protease TMPRSS2, and several RAAS genes. Moreover, we show that interaction of the ACE2 receptor with the SARS-CoV-2 spike protein activates the Nlrp3 inflammasome in human cells, which, if hyperactivated, may lead to cell death by pyroptosis, as is well known. Based on this mechanism, early-development human stem cells residing in adult tissues may be affected by SARS-CoV-2, which could lead to long-term defects in tissue/organ regeneration. We also report that ACE2 is expressed on the surface of murine bone marrow-derived VSELs and HSCs, although it is known that murine cells are not infected by SARS-CoV-2. Finally, human and murine VSELs express several RAAS genes, which sheds new light on their role in the specification of early-development stem cells.

## Materials and Methods

### Mice and Umbilical Cord Blood

Pathogen-free, 6–8-week-old female C57BL/6 J mice were purchased from the Jackson Laboratory (Bar Harbor, ME; USA) at least 2 weeks before experiments. Animal studies were approved by the Animal Care and Use Committee of the University of Louisville (Louisville, KY, USA) and Warsaw Medical University (Warsaw, Poland). Clinical-grade UCB research units were shipped from the Cleveland Cord Blood Center. This study was performed in accordance with the guidelines and approval of the Institutional Review Board at the University of Louisville School of Medicine (Louisville, Kentucky).

### Murine Bone Marrow-Derived Mononuclear Cells (BMMNCs)

Cells were obtained by flushing experimental mouse tibias and femurs. Red blood cells (RBCs) were removed by lysis in BD Pharm Lyse buffer (BD Biosciences, San Jose, CA, USA), washed, and resuspended in appropriate media.

### Sorting Strategy for VSELs and HSCs from Human Umbilical Cord Blood (UCB)

**Fig. 1:**
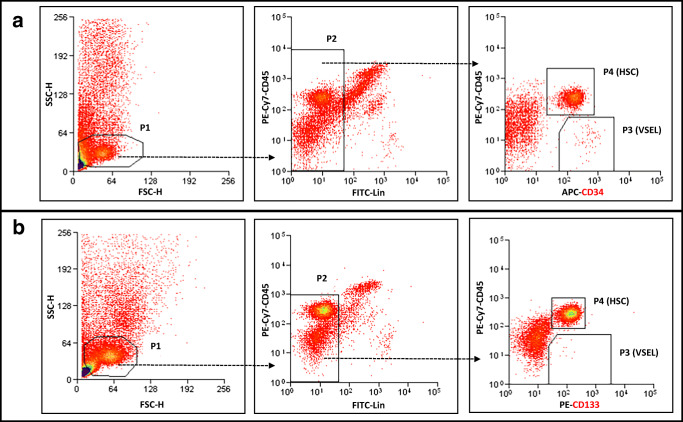
Gating strategy for sorting human CD34^+^ and CD133^+^ VSELs and HSCs by FACS. UCB-derived VSELs and HSCs were isolated from human UCB mononuclear cells (MNCs) following magnetic isolation of CD34 or CD133-positive cells and immunostaining. UCB derived populations of MNCs was visualized by dot plot showing forward scatter (FSC) vs. side scatter (SSC) signals, which are related to the size and granularity/complexity of the cells. Cells from region P1 were further analyzed for CD45 and Lin expression. The population of CD45^+^/Lin^−^ objects was included in region P2. Cells from region P2 were analyzed for CD45 and CD34 expression and subsequently sorted into Lin^−^/CD34^+^/CD45^−^ cell (VSEL, region P3) and Lin^−^/CD34^+^/CD45^+^ cell (HSC, region P4) subpopulations (Panel **a**) or CD45 and CD133 expression and sorted into Lin^−^/CD133^+^/CD45^−^ cell (VSEL, region P3) and Lin^−^/CD133^+^/CD45^+^ cell (HSC, region P4) subpopulations (Panel **b**)

VSELs (Lin^−^/CD34^+^/CD45^−^ or Lin^−^/CD133^+^/CD45^−^) and HSCs (Lin^−^/CD34^+^/CD45^+^ or Lin^−^/CD133^+^/CD45^+^) were isolated from human umbilical cord blood (hUCB) samples from healthy full-term newborns. Samples were obtained through a partnership with the Cleveland Cord Blood Bank Center under IRB approval for scientific research purposes. Briefly, mononuclear cells (MNCs) were isolated from hUCB samples by density-gradient centrifugation on a Ficoll–Paque gradient (ρ = 1.077 g/mL; GE Healthcare). MNCs were then labelled with anti-CD34 or anti-CD133 MicroBeads (Miltenyi Biotec) and separated on magnetic columns (Miltenyi Biotec). The cell population of CD34^+^ or CD133^+^-enriched cells was stained with fluorescence-labeled antibodies (all Becton Dickinson) for the hematopoietic lineage marker (Lin), CD45, and CD34 or CD133. The following murine anti-human antibodies were employed for staining: anti-CD235a (FITC; clone GA-R2 [HIR2]), anti-CD2 (FITC; clone RPA-2.10), anti-CD3 (FITC; clone UCHT1), anti-CD14 (FITC; clone M5E2), anti-CD16 (FITC; clone 3G8), anti-CD19 (FITC; clone, HIB19), anti-CD24 (FITC; clone ML5), anti-CD56 (FITC; clone NCAM16.2), anti-CD66b (FITC; clone G10F5), anti-CD45 (PE–Cy7; clone HI30), and anti-CD34 (APC; clone 581) or anti-CD133 (PE; clone AC133). Staining was performed in RPMI-1640 medium supplemented with 2% FBS on ice for 30 min. The cells was subsequently washed, resuspended in RPMI1640 containing 2% FBS, and sorted using a MoFlo XPD cell sorter (Beckman Coulter) as a highly purified population of Lin^−^/CD34^+^/CD45^−^ VSELs and Lin^−^/CD34^+^/CD45^+^ HSC using the strategy presented in Fig. 1a or Lin^−^/CD133^+^/CD45^−^ VSELs and Lin^−^/CD133^+^/CD45^+^ HSCs using the strategy presented in Fig. 1b.

### Sorting Strategy for VSELs and HSCs from Murine Bone Marrow

Lin-/Sca-1^+^/CD45^−^ cells (VSELs) and Lin^−^/Sca-1^+^/CD45^+^ cells (HSCs) were isolated from the BM of adult C57BL/6 mice (five weeks old; Jackson Laboratory, USA). Bone marrow was flushed from the cavities of tibias and femurs, and the cell suspension was collected and filtered through a 70-μm strainer (BD Bioscience). The population of total nucleated cells (TNCs) was obtained after lysis of RBCs using 1× BD Pharm Lyse buffer (BD Pharmingen, USA), washed with phosphate-buffered saline (DPBS; w/o Ca^2+^ or Mg^2+^; Life Technologies), and resuspended in PBS containing 2% fetal bovine serum (FBS, Thermo Fisher Scientific). Staining for VSELs and HSCs was performed with fluorescence-labeled antibodies for CD45, the hematopoietic lineage marker (Lin), and Sca-1. The following anti-mouse antibodies were employed for staining: rat anti-CD45 (allophycocyanin [APC]–Cy7; clone 30-F11), anti-CD45R/B220 (phycoerythrin [PE]; clone RA3-6B2), anti-Gr-1 (PE; clone RB6-8C5), anti-TCRαβ (PE; clone H57–597), anti-TCRγδ (PE; clone GL3), anti-CD11b (PE; clone M1/70), anti-Ter119 (PE; clone TER-119), and anti-Ly-6A/E (also known as Sca-1; biotin; clone E13–161.7, with streptavidin conjugated to PE–Cy5). All antibodies were obtained from BD Biosciences. Staining was performed in PBS with 2% FBS on ice for 30 min. The cells were subsequently washed, resuspended in DMEM plus 2% FBS, and sorted using a FACS Melody cell sorter (Becton Dickinson). The Sca-1^+^Lin^−^CD45^−^ cells (VSELs) and Sca-1^+^Lin^−^CD45^+^ cells (HSCs) were isolated according to the gating and sorting strategy described in Fig. 4.

### Detection of ACE2 on the Surface of Human Cells by FACS

ACE2 expression was detected on human UCB-derived VSELs and HSCs. Briefly, mononuclear cells (MNCs) were isolated from hUCB samples by density-gradient centrifugation on a Ficoll–Paque gradient (ρ = 1.077 g/mL; GE Healthcare). MNCs were then labelled with anti-CD34 or anti-CD133 MicroBeads (Miltenyi Biotec) and separated on magnetic columns (Miltenyi Biotec). The cell population of CD34^+^ or CD133^+^-enriched cells was stained with fluorescence-labeled antibodies for the hematopoietic lineage marker (Lin – listed in VSELs and HSCs sorting method), CD45 (PE–Cy7; clone HI30, Becton Dickinson), and CD34 (APC; clone 581, Beckman Dickinson) or CD133 (APC; clone AC133, Beckman Dickinson). Additionally, cells were stained with ACE2 antibody (PE, clone E-11, Santa Cruz). Staining was performed in RPMI-1640 medium supplemented with 2% FBS on ice for 30 min. The cells were subsequently washed, resuspended in RPMI1640 containing 2% FBS, and analyzed on BD LSR II flow cytometer (Becton Dickinson).

### Real-Time RT-PCR

VSELs, HSCs, and MNCs FACS-sorted from murine bone marrow as well as VSELs, HSCs, and MNCs isolated from UCB were lysed, and then total RNA was isolated using the miRNeasy Mini kit (Qiagen, CA). cDNA was prepared from RNA template using the iScript cDNA Synthesis kit (Bio-Rad) according to the manufacturer’s recommendations. Real-time PCR was performed using a CFX384 Touch™ Real-Time PCR detection system (Bio-Rad) with iTaq Univer SYBR Green Supermix (Bio-Rad) reagent. All PCRs were performed using the following conditions: pre-denaturation at 95 °C for 3 min, 40 cycles of denaturation at 95 °C for 10s, and annealing at 60 °C for 60 s. A melting curve was generated to confirm primer specificity and to avoid the possibility of amplifying DNA contamination. The mRNA levels of target genes were normalized to the β2 microglobulin mRNA level. The average cycle threshold (Ct) value of three technical replicates was used for the comparative Ct (ΔΔCt) method. The primer sequences used for RT-qPCR are listed in Tables 1 and 2.

**Table 1:** List of primer sequences used for Real-Time qPCR analysis of cells isolated from human umbilical cord blood.

Name of the gene	Primer	Sequence
*β2M*	forward	5’-TGACTTTGTCACAGCCCAAGATA-3′
	reverse	5’-AATGCGGCATCTTCAAACCT-3′
*ACE2*	forward	5’-TCCGTCTGAATGACAACAGC -3′
	reverse	5’-CACTCCCATCACAACTCCAA-3′
*AT1*	forward	5’-CAGCCAGCGTCAGTTTCAACC -3′
	reverse	5’-GGCTACAAGCATTGTGCGTCG −3′
*AT2*	forward	5’-GACCTTCCTGGATGCTCTGG −3′
	reverse	5’-CGCAGCTGTTGGTGAATCCC -3′
*MAS*	forward	5’-GTCCAGCACCATCTTGGTCG −3’
	reverse	5’-GGAGTCTCATGGGCATAGCG −3’
*NLRP3*	forward	5’-GTGTGGGACTGAAGCACCTG −3’
	reverse	5’-GTCTCCCAAGGCATTCTCCC -3’
*IL-1 beta*	forward	5’-TGCCCACAGACCTTCCAGGA −3’
	reverse	5’-CGGAGCGTGCAGTTCAGTGA −3’
*IL-18*	forward	5’-CGGCCTCTATTTGAAGATATGAC -3’
	reverse	5’-CCATACCTCTAGGCTGGCTA −3’
*RENIN*	forward	5’-ATCCACCTTGCTCTGTGAAG −3’
	reverse	5’-CCTGAAATACATAGTCCGCG −3’
*CMA1*	forward	5’-GTGTGGGCAATCCCAGGAAG −3’
	reverse	5’-CCGACCGTCCATAGGATACG −3’
*AIM2*	forward	5’-AACGTGCTGCACCAAAAGTC -3’
	reverse	5’-ACGTGAAGGGCTTCTTTGCT −3’
*ASC*	forward	5’-CCCAAGCAAGATGCGGAAGC -3’
	reverse	5’-TTAGGGCCTGGAGGAGCAAG −3’
*NLRP1*	forward	5’-GATTGAGGGCAGGCAGCACA −3’
	reverse	5’-TCTGGTGACCTTGAGGACGG −3’

**Table 2:** List of primer sequences used for Real-Time qPCR analysis of murine cells isolated from bone marrow

Name of the gene	Primer	Sequence
*β2M*	forward	5’-CATACGCCTGCAGAGTTAAGCA-3′
	reverse	5’-GATCACATGTCTCGATCCCAGTAG-3′
*ACE2*	forward	5’-TTCTGGGCAAACTCTATGCTG-3′
	reverse	5’-CTCGTGATGGGCTGTCAAG-3′
*AT1*	forward	5’-GCTTCCTGTTCCCTTTCCTA-3’
	reverse	5’-TCATTTCTTGGCTTGTTCTTCT-3’
*AT2*	forward	5’-GATCTGGTGCAGTTACATCTCAG-3’
	reverse	5’-CTTACTCAGCTCCCGCATG-3’
*AT3*	forward	5’-ACATTCTGGGCTTCGTGTTC-3’
	reverse	5’-TGTCATCATTCCTTGGCGTA-3’
*MAS*	forward	5’-TTCAGTTGGAAGCGGAGTTT-3’
	reverse	5’-TGGCACAGAGACCTGCTACA-3’
*TMPRSS2*	forward	5′-GGATTGTGGGTGGATTGAA-3’
	reverse	5’-GCTGCTGAGGGGTTCTTC-3’
*REN*	forward	5’-GCGAGATTGGCATCGGTA-3’
	reverse	5’-TCCCACAAGCAAGGTAGAGG-3’
*AGT*	forward	5’-AGCATCTCGGTGTCTGTGC-3’
	reverse	5’-AGCAGGGTGGCTCTCTCAC-3’
*ACE*	forward	5’-GGTAGTGCCTTTCCCAGACA-3’
	reverse	5’-GACTCCGCCCAGAACTCAG-3’
*NLRP3*	forward	5’-ACCAGCCAGAGTGGAATGAC-3’
	reverse	5’-ATGGAGATGCGGGAGAGATA-3’
*CASP1*	forward	5’-GCTTTCTGCTCTTCAACACC-3’
	reverse	5’-AAAATGTCCTCCAAGTCACAAG-3’
*IL-1 beta*	forward	5’-AGTTGACGGACCCCAAAAG-3’
	reverse	5’-CTTCTCCACAGCCACAATGA-3’
*IL-18*	forward	5’-ACAACTTTGGCCGACTTCAC-3’
	reverse	5’-GTCTGGTCTGGGGTTCACTG-3’
*GSDM*	forward	5’-CTGGGTCTTGCTAGAAGAATGTGG-3’
	reverse	5’-CTGGCCTAGACTTGACAATAGGAAC-3’
*HMGB1*	forward	5’-GGAGGAGCACAAGAAGAAGC-3’
	reverse	5’-GGGGGATGTAGGTTTTCATTT-3’

### RT-PCR Analysis

Total RNA was isolated using the RNeasy Mini kit, including treatment with DNase I (both from Qiagen Inc., Germantown, MD, USA). The purified mRNA (50 ng) was afterwards reverse transcribed into cDNA using the First Strand cDNA Synthesis kit (Thermo Scientific, Waltham, MA, USA) according to the manufacturer’s instructions and using a mixture of oligo(dT) and random hexamers. Amplification of the synthesized cDNA fragments was carried out using TaKaRa Taq™ DNA Polymerase (Takara Bio USA, Inc.) and sequence-specific ACE2 primers with 1 cycle of 8 min at 95 °C; 2 cycles of 2 min at 95 °C, 1 min at 60 °C, and 1 min at 72 °C; 45 cycles of 30 s at 95 °C, 1 min at 60 °C, 1 min at 72 °C; and 1 cycle of 10 min at 72 °C.

### Exposure of Cells to SARS-CoV-2 Spike Protein and Angiotensin Angiotensin Fragment 1–7

UCB-derived HSCs and VSELs were plated in 96-well plates and stimulated with NCP-CoV (2019-nCoV) spike protein (S1 + S2 ECD, with His-tag; Sino Biological) at a concentration of 10 nM. After 16 h of incubation, the cells were lysed, and total RNA was isolated for qRT-PCR analysis of *Nlrp3, ASC, AIM2, IL1β, IL18,* and *Nlrp1* expression. In some experiments UCB-derived HSC were plated in 96-well plates and stimulated with NCP-CoV (2019-nCoV) spike protein (S1 + S2 ECD, with His-tag; Sino Biological) at a concentration of 10 nM alone or together with angiotensin Fragment 1–7 (Millipore Sigma) at a concentration of 300 μg/ml.

### Statistical Analysis

All results are presented as mean ± SD. Statistical analysis of the data was done using Student’s t test for unpaired samples, with *p* ≤ 0.05 considered significant.

## Results

**Fig. 2:**
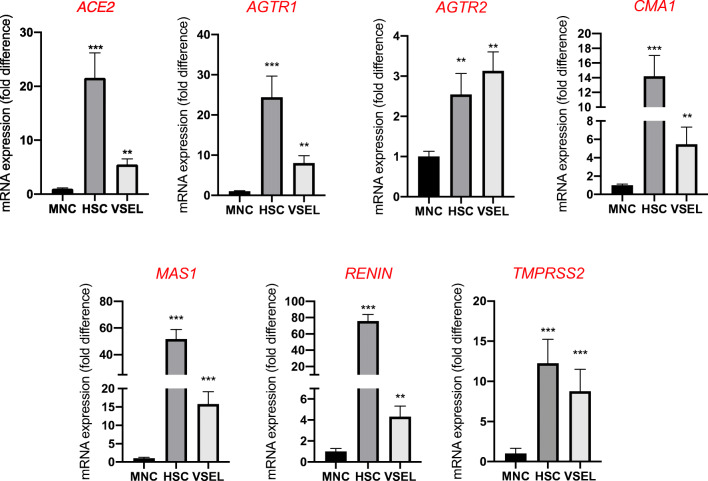
Expression of SARS-CoV-2 entry receptors and selected RAAS genes in purified human VSELs and HSCs. Expression of ACE2, AGTR1, AGTR2, MAS1, CMA1, RENIN and TMPRSS2 mRNAs in UCB MNC and UCB purified VSELs and HSCs as measured by RT-PCR. To evaluate relative expression, comparative ΔCT method was employed. Results are combined from three independent purification of UCB VSELs and HSCs. Results are presented as mean ± SEM. *P < 0.05, **P < 0.01, ***P < 0.001

Like HSCs, highly purified human VSELs express mRNA for ACE2, TMPRSS2, and RAAS peptides and receptors as well as components of the Nlrp3 inflammasome complex. We sorted very small CD34^+^Lin^−^CD45^−^ cells (VSELs) and CD34^+^Lin^−^CD45^+^ cells (HSCs) from UCB by FACS (Fig. 1) and phenotyped them by real-time PCR for expression of mRNAs for the ACE2 entry receptor for SARS-CoV-2, the spike protein-processing enzyme TIMPRSS2, the receptors for Ang II (AT_1_R and AT_2_R), and the Ang (1–7) receptor (MasR, Fig. 2). We found that all of these receptors were expressed in human VSELs and HSCs. In addition, both populations of cells expressed the *CMA1* gene, encoding chymase, a chymotryptic serine proteinase, which is also involved in the processing of Ang I into Ang II. Interestingly, we also detected expression of renin, an enzyme that processes conversion of angiotensinogen into Ang I, indicating that RAAS is involved in regulating the biology of both types of cells.

**Fig. 3:**
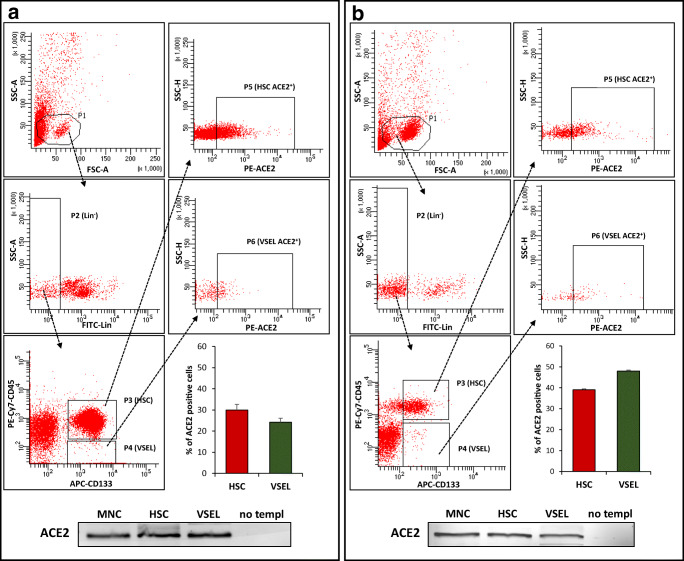
Expression of ACE2 on human CD34^+^ and CD133^+^ VSELs and CD34^+^ and CD133^+^ HSCs. Expression of ACE2 was evaluated on human UCB-derived VSELs and HSC by FACS and RT-PCR. Panel **a**. Detection of ACE2 on Lin^−^/CD34^+^/CD45^−^ VSELs (second raw) and Lin^−^/CD34^+^/CD45^+^ HSCs (first raw). Panel **b**. Detection of ACE2 on Lin^−^/CD133^+^/CD45^−^ VSELs (second raw) and Lin^−^/CD133^+^/CD45^+^ HSCs (first raw). Results from flow cytometry are presented as a percentage of ACE2^+^ cells. The data represent the mean value ± SEM for two independent experiments (upper part of Panel **a** and **b**). The expression of ACE2 gene was detected in purified mRNA from sorted human VSELs and HSCs by reverse transcription polymerase chain reaction (RT-PCR). Samples containing only water instead of cDNA and samples without reverse transcriptase were used in each run as negative controls. Representative agarose gels of the RT-PCR amplicon are shown (lower part of Panel **a** and **b**)

Importantly, expression of SARS-CoV-2 entry receptor ACE2 was subsequently confirmed at the protein level by FACS on both CD34^+^ and CD133^+^ VSELs and HSCs (Fig. 3a and b). Expression of ACE2 was slightly higher on the surface of CD133^+^ VSELs (~50%) than on CD34^+^ VSELs (~20%). At the same time CD34^+^ HSC ~30% expressed ACE2 and CD133^+^ HSC ~40%.

**Fig. 4:**
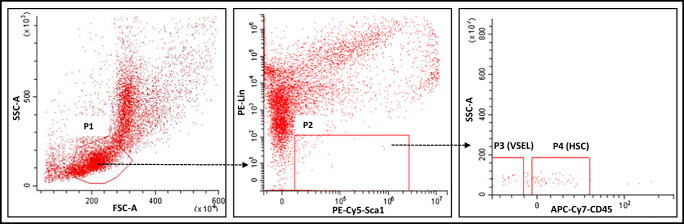
Gating strategy for sorting murine VSELs and HSCs by FACS. BM-derived VSELs and HSCs were isolated from murine BM total nucleated cells (TNCs) following immunostaining for Sca-1, CD45, and the hematopoietic lineage marker (Lin). BM-derived TNCs were visualized by dot plot showing forward scatter (FSC) vs. side scatter (SSC) signals, which are related to the size and granularity/complexity of cells. Cells from region P1 are further analyzed for Sca-1 and Lin expression. The population of Sca-1^+^/Lin^−^ objects was included in region P2 and subsequently sorted into CD45^−^ and CD45^+^ subpopulations based on CD45 marker expression. Region P3 shows Sca-1^+^/Lin^−^/CD45^−^ cells (VSELs). Region P4 shows Sca-1^+^/Lin^−^/CD45^+^ cells (VSELs)

**Fig. 5:**
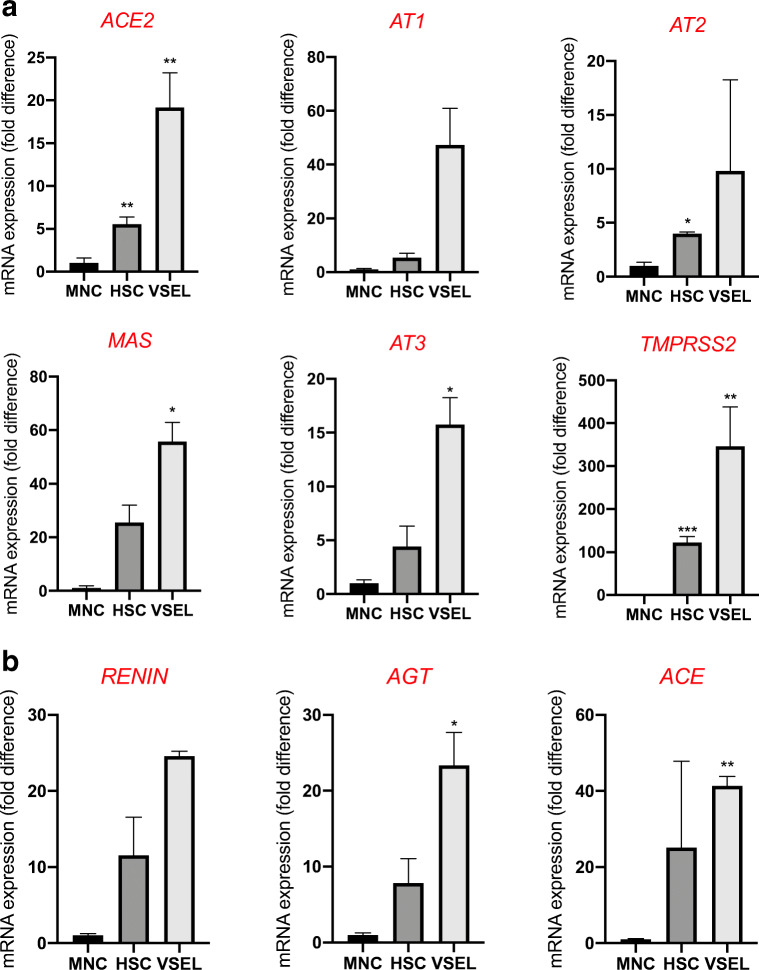
The relative expression of mRNAs for ACE2, RAAS peptides and receptors, and components of the Nlrp3 inflammasome in highly purified murine bone marrow-derived VSELs and HSCs. Real-time PCR quantitation of FACS-sorted murine VSELs and HSCs in comparison with mononuclear cells; *P < 0.05, **P < 0.01, ***P < 0.001. In order to evaluate relative expression, the comparative ΔCT method was employed. Results are presented as mean ± SEM. Panel **a**. Differences in the expression of mRNAs for angiotensin-converting enzyme 2 (ACE2), the type 1 angiotensin II receptor (AT1), the type 2 angiotensin II receptor (AT2), the proto-oncogene Mas (MAS), the type 3 angiotensin II receptor (AT3), and transmembrane protease 2 (TMPRSS2). Panel **b**. Differences in the expression of mRNAs for renin (REN), angiotensinogen (AGT), and angiotensin-converting enzyme (ACE)

Highly purified murine VSELs and HSCs express mRNAs for ACE2, TMPRSS2, and RAAS peptides and receptors as well as components of the Nlrp3 inflammasome complex. Although murine cells are not infected by SARS-CoV-2, we became interested in whether the murine counterparts of human VSELs and murine HSCs express the virus-entry receptor and components of the RAAS. To address this question, we purified murine bone marrow-derived VSELs and HSCs by FACS sorting (Fig. 4), and the mRNA expression for all of these genes was evaluated by real-time PCR. Figure 5a shows that murine VSELs express, at much higher levels than HSCs and mononuclear cells (MNCs), mRNAs for ACE2, AT_1_R, AT_2_R, AT_3_R, MAS, and transmembrane protease 2 (TMPRSS2), which in human cells primes the SARS-CoV-2 spike protein (S) for better interaction with ACE2. Interestingly, Fig. 5b demonstrates that murine VSELs and HSCs highly express mRNAs for angiotensinogen, renin, and the ACE receptor, which indicates that these cells may be regulated by intrinsic RAAS. Because they also express AT_1_R and MasR, they may respond to Ang II and Ang (1–7) stimulation.

Stimulation of human HSCs and VSELs with SARS-CoV-2 spike protein (S) activates the Nlrp3 inflammasome. We have proposed that hyperactivation of the Nlrp3 inflammasome is the culprit in the induction of cytokine storms in SARS-CoV-2-infected patients, leading to multi-organ damage. There are different mechanisms that may lead to this problem, involving extensive stimulation of AT_1_R by Ang II or enhanced activity of the complement cascade, which recognizes viral proteins by the lectin pathway, immune complexes by the classical pathway, and responds to Toll-like receptor activation by activating the alternative pathway. We recently hypothesized that the Nlrp3 inflammasome is activated after stimulation of ACE2 by viral spike protein (S).

**Fig. 6:**
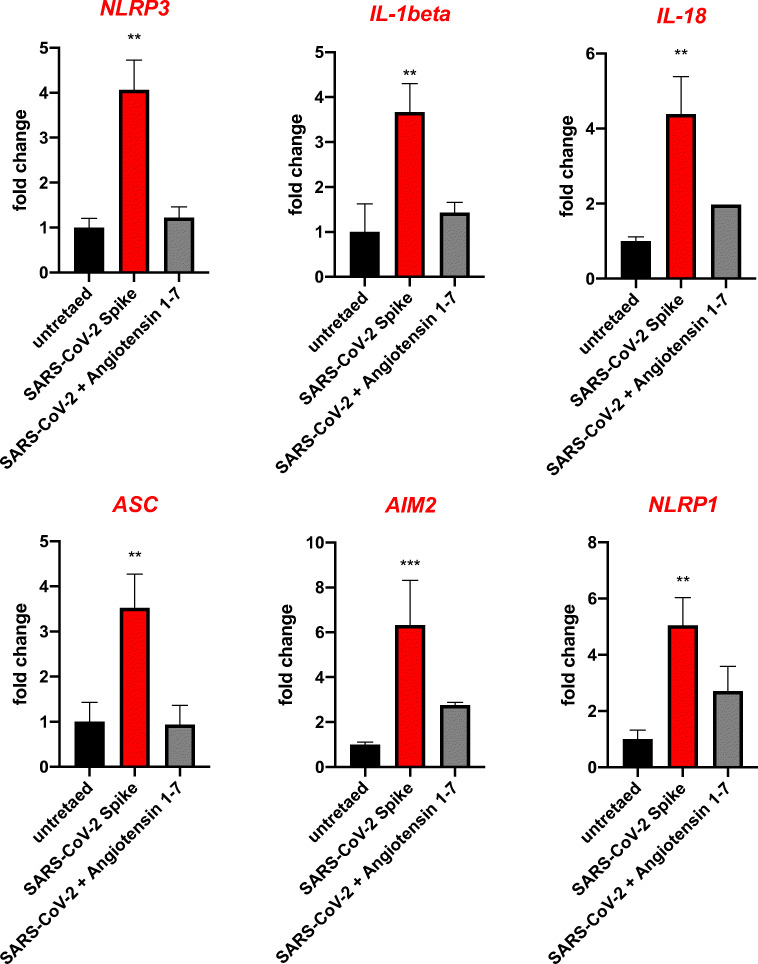
Human CD34^+^ HSC activate Nlrp3 inflammasome in response to SARS-Cov-2 spike protein. Effect of NCP-CoV (2019-nCoV) Spike protein (S1 + S2 ECD, His tag) and Angiotensin 1–7 on the expression of inflammasome related genes. Real-time PCR quantitation of FACS sorted human UCB derived HSCs in comparison to mononuclear cells; *P < 0.05, **P < 0.01, ***P < 0.001. In order to evaluate relative expression, comparative ΔCT method was employed. Results are presented as mean ± SEM. Differences in the expression of mRNAs for NLRP3, AIM2, ASC, IL-1beta, IL-19 and NLRP1 after 16 h exposure to SARS-CoV-2 Spike alone and in the presence of Angiotensin 1–7

**Fig. 7:**
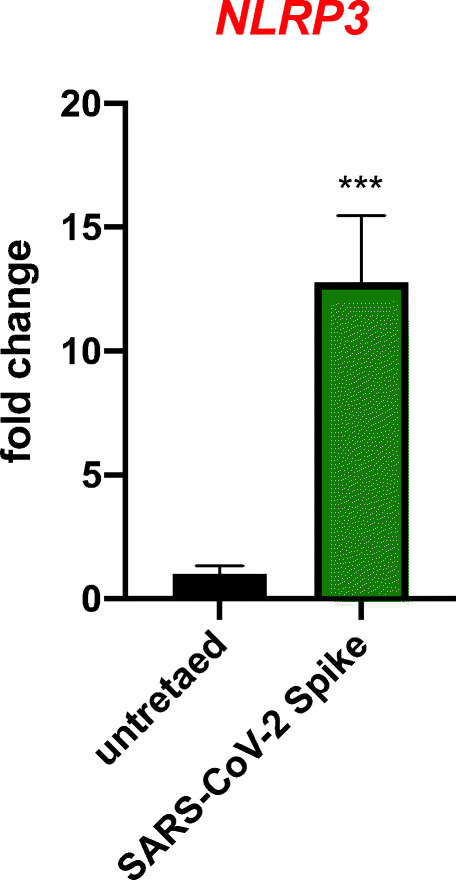
Human CD34^+^ VSELs activate Nlrp3 inflammasome in response to SARS-Cov-2 spike protein. Effect of NCP-CoV (2019-nCoV) Spike protein (S1 + S2 ECD, His tag) on the expression of inflammasome related genes. Real-time PCR quantitation of FACS sorted human UCB derived VSELs in comparison to mononuclear cells; *P < 0.05, **P < 0.01, ***P < 0.001. In order to evaluate relative expression, comparative ΔCT method was employed. Results are presented as mean ± SEM. Differences in the expression of mRNAs for NLRP3 after 16 h exposure to SARS-CoV-2 Spike protein

To test this hypothesis, we exposed human UCB-isolated CD34^+^lin^−^CD45^+^ and CD133^+^lin^−^CD45^+^ HSCs to recombinant S protein for 16 h, and activation of the Nlrp3 inflammasome was evaluated for changes in mRNA expression of Nlrp3, IL-1β, IL-18, and ASC. We also evaluated changes in expression of mRNAs for two other inflammasomes, Aim2 and Nlrp1. We show for the first time that exposure of UCB HSCs to spike protein enhances transcription of all mRNAs evaluated in our studies. At the same time activation of Nlrp3 inflammasome in response to spike protein was attenuated in presence of Ang 1–7 (Fig. 6). We also detected elevated levels of secreted IL-1β, as determined by ELISA, in the conditioned media from cells stimulated by spike protein, which is an important indicator of Nlrp3 inflammasome activation. Importantly, we exposed human UCB-purified VSELs to spike protein, and as shown in Fig. 7 we found upregulation of Nlrp3 mRNA.

**Fig. 8:**
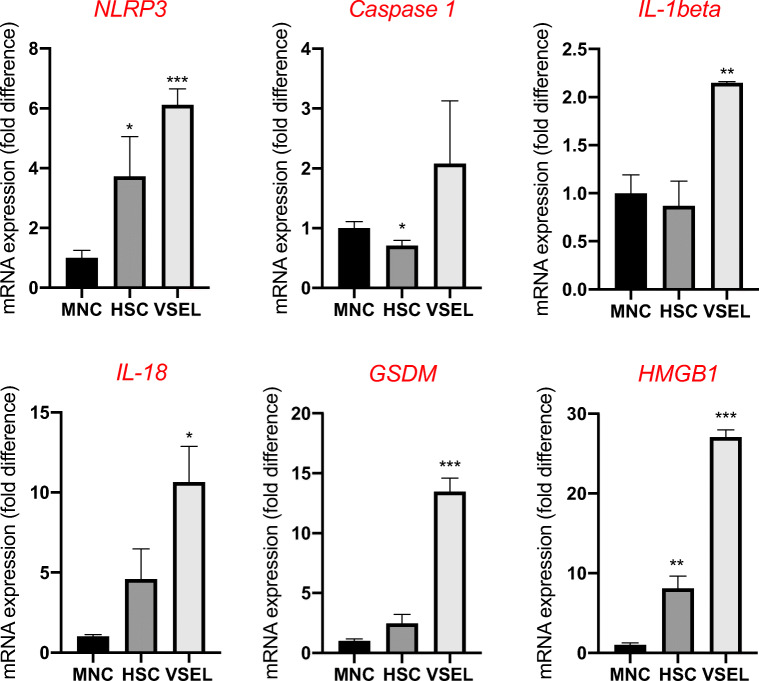
Murine VSELs express mRNAs for elements of the Nlrp3 inflammasome. Real-time PCR quantitation of FACS-sorted murine VSELs and HSCs in comparison with mononuclear cells; *P < 0.05, **P < 0.01, ***P < 0.001, ****P < 0.0001. In order to evaluate relative expression, the comparative ΔCT method was employed. The relative quantity of target gene was normalized to the endogenous β2 microglobulin gene. Results are presented as mean ± SEM. Graphs represent differences in the expression of mRNAs for NACHT, LRR, and PYD domain-containing protein 3 (NLRP3); caspase 1 (CASP1); interleukin 1β (IL-1β); interleukin 18 (IL-18); gasdermin (GSDM); and high-mobility group protein B1 (HMGB1)

Interestingly, murine VSELs also highly express mRNAs for elements of the Nlrp3 inflammasome and RAAS genes (Fig. 8). Nevertheless, they are not infectable by SARS-CoV-2. However, they may be regulated by RAAS similarly as have been proposed for putative hemangioblasts [41].

## Discussion

The most important message of this report is that human early-development stem cells deposited in adult tissues, known as very small embryonic-like stem cells (VSELs), like specified HSCs or EPCs, express on their surface the ACE2 receptor for entry of SARS-CoV-2 as well as the entry-facilitating protease TMPRSS2. In addition, these cells express AT_1_R, which indicates that they can be challenged by Ang II stimulation. We also report for the first time that the virus-derived recombinant spike protein (S) activates the Nlrp3 inflammasome in human VSELs and HSCs.

Our results are highly relevant to the potential long-term effects of SARS-CoV-2 infection, as the virus may damage VSELs by *i)* direct entry and cell lysis or *ii)* induction of cell death by pyroptosis due to hyperactivation of the Nlrp3 inflammasome in response to S protein or to excessive Ang II stimulation by AT_1_R. Nlrp3 inflammasome-induced cell death by pyroptosis is a result of activation of caspase 1, the release of IL-1β and IL-18, the perturbation of mitochondrial function, the creation of pores in the cell membrane by gasdermin D, and the release of several alarmines or danger-associated molecular pattern molecules (DAMPs) that subsequently amplify an uncontrolled immune response [16, 42–44]. This response involves secretion from other cells of several pro-inflammatory cytokines such as. IL-6 or TNF-α and mediators as well as activation of the complement and coagulation cascades [45–47]. If uncontrolled, this process may end in a cytokine storm and fatal organ damage.

It is well known that the innate immune response and activation of the Nlrp3 inflammasome are important defense mechanisms during the first days of infection, until acquired immunity responds with the production of antibodies. However, on the other hand hyperactivation of this intracellular protein complex may induce a cytokine storm, with detrimental effects, leading to multi-organ failure. We have proposed three scenarios for how this could happen. First, the SARS-CoV-2 spike protein (S), after binding to cell surface-expressed ACE2, directly triggers Nlrp3 inflammasome activation. In fact, we show for the first time that S protein binding to ACE2 on human cells contributes to activation of the Nlrp3 inflammasome. Importantly, we found that activation of the Nlrp3 inflammasome in human cells after the interaction of ACE2 with the SARS-CoV-2 S protein is ameliorated in the presence of Ang (1–7). Second, as previously reported, after engaging the AT1 receptor, Ang II activates the Nlrp3 inflammasome in lung, kidney cells, and cardiomyocytes, and excessive activation of the Ang II–AT_1_R axis in these cells may lead to pyroptosis [12, 48, 49]. Since, as shown in our studies, both VSELs and HSCs highly express AT_1_R, there is *i)* an increase in Ang II activity in patients infected with SARS-CoV-2 and *ii)* a lack of protective Ang (1–7)–Mas signaling due to blockade and downregulation of ACE2 by viral proteins, which may hyperactivate the Ang II–AT_1_R axis. Third, recognition and interaction of the complement cascade with SARS-CoV-2 releases several potent cleavage fragments, including C3a and C5a anaphylatoxins as well as the C5bC9 membrane attack complex (MAC), which may also directly trigger activation of the Nlrp3 inflammasome in target cells including population of stem cells.

Therefore, the potential damage of VSELs after virus entry or exposure to Nlrp3 inflammasome hyperactivating mediators may have a negative effect on the regenerative potential of SARS-CoV-2-infected individuals and create potential long-term health problems in survivors. This, however, will require further study. In support of this possibility, ACE2 function and Ang (1–7)–MasR signaling, which are perturbed by SARS-CoV-2 infection, play a role in the proliferation of several stem cell types, including HSCs, EPCs, and skeletal muscle cells [6, 15, 50]. Moreover, since several complications from endothelium have been already reported, it is important to assess the effects of infection on hematopoiesis and lymphopoiesis and also take into account damage of more primitive precursors of these cells that are VSELs.

Another important observation is that RAAS mediators may play a role in the development of VSELs. As mentioned in the introduction, the role of RAAS in the development of putative hemangioblasts has been reported in several old publications [39, 41, 51, 52]. These cells were purified for example from human para-aortic splanchnopleura as CD143^+^, CD34^−^, and CD45^−^ population [53]. They expressed the ACE receptor (CD143), however they were not evaluated for the expression of SARS-Cov-2 entry receptor ACE2. In our studies human VSELs, which may correspond to hemangioblasts in in vitro assays, are present among the population of human ACE2^+^, very small CD133^+^CD34^+^Lin^−^CD45^−^ cells. These cells, which have been isolated from adult bone marrow, mobilized peripheral blood, and umbilical cord blood, according to several independent reports, have been proposed to serve in postnatal tissues as a backup stem cell population involved in tissue or organ rejuvenation [26, 33–35, 54]. Importantly in appropriate experimental models they can become specified into functional HSCs and EPCs.

In conclusion, since we still do not have an effective SARS-CoV-2 vaccine in hand, the results presented in our current work suggest that inhibition of the Nlrp3 inflammasome by the small-molecule inhibitor MCC950 or application of Nlrp3 inflammasome inhibitors, such as Ang (1–7) or heme oxygenase 1 activators, could find potential clinical applications to prevent onset of a cytokine storm and cell pyroptosis [23]. In further support of this possibility, encouraging results have already been obtained in employing antibodies against a Nlrp3 inflammasome-activation product, IL-1β. Finally, based on our results demonstrating ACE2 expression on the surface of VSELs, further studies of infection with live virus will be needed to address whether the virus may enter these cells. Finally, the expression of several genes involved in RAAS in VSELs raises the possibility that this system is involved in the development and specification of early-development stem cells.

## References

[1] Fleming I (2006). Signaling by the angiotensin-converting enzyme. Circ Res.

[2] Gheblawi M, Wang K, Viveiros A, Nguyen Q, Zhong JC, Turner AJ, Raizada MK, Grant MB, Oudit GY (2020). Angiotensin-converting enzyme 2: SARS-CoV-2 receptor and regulator of the renin-angiotensin system: Celebrating the 20th anniversary of the discovery of ACE2. Circ Res.

[3] Schwacke JH, Spainhour JC, Ierardi JL, Chaves JM, Arthur JM, Janech MG (2013). Network modeling reveals steps in angiotensin peptide processing. Hypertension.

[4] Li W, Moore MJ, Vasilieva N, Sui J, Wong SK, Berne MA (2003). Angiotensin-converting enzyme 2 is a functional receptor for the SARS coronavirus. Nature.

[5] Kuba K, Imai Y, Rao S, Gao H, Guo F, Guan B, Huan Y, Yang P, Zhang Y, Deng W, Bao L, Zhang B, Liu G, Wang Z, Chappell M, Liu Y, Zheng D, Leibbrandt A, Wada T, Slutsky AS, Liu D, Qin C, Jiang C, Penninger JM (2005). A crucial role of angiotensin converting enzyme 2 (ACE2) in SARS coronavirus-induced lung injury. Nat Med.

[6] Joshi S, Wollenzien H, Leclerc E, Jarajapu YP (2019). Hypoxic regulation of angiotensin-converting enzyme 2 and mas receptor in human CD34(+) cells. J Cell Physiol.

[7] Rodgers KE, Xiong S, Steer R, diZerega GS (2000). Effect of angiotensin II on hematopoietic progenitor cell proliferation. Stem Cells.

[8] Hoffmann M, Kleine-Weber H, Schroeder S, Kruger N, Herrler T, Erichsen S (2020). SARS-CoV-2 cell entry depends on ACE2 and TMPRSS2 and is blocked by a clinically proven protease inhibitor. Cell.

[9] Chappell MC, Pirro NT, Sykes A, Ferrario CM (1998). Metabolism of angiotensin-(1-7) by angiotensin-converting enzyme. Hypertension.

[10] Singh, K. D., & Karnik, S. S. (2016). Angiotensin receptors: Structure, function, signaling and clinical applications. *J Cell Signal, 1*(2). 10.4172/jcs.1000111 PMC4976824 27512731

[11] Ren XS, Tong Y, Ling L, Chen D, Sun HJ, Zhou H, Qi XH, Chen Q, Li YH, Kang YM, Zhu GQ (2017). NLRP3 gene deletion attenuates angiotensin II-induced phenotypic transformation of vascular smooth muscle cells and vascular remodeling. Cellular Physiology and Biochemistry.

[12] Zhao M, Bai M, Ding G, Zhang Y, Huang S, Jia Z, Zhang A (2018). Angiotensin II stimulates the NLRP3 Inflammasome to induce Podocyte injury and mitochondrial dysfunction. Kidney Dis (Basel).

[13] Chen IY, Moriyama M, Chang MF, Ichinohe T (2019). Severe acute respiratory syndrome coronavirus Viroporin 3a activates the NLRP3 Inflammasome. Frontiers in Microbiology.

[14] Gan W, Ren J, Li T, Lv S, Li C, Liu Z, Yang M (2018). The SGK1 inhibitor EMD638683, prevents angiotensin II-induced cardiac inflammation and fibrosis by blocking NLRP3 inflammasome activation. Biochim Biophys Acta Mol Basis Dis.

[15] Jarajapu, Y. P. (2020). Targeting ACE2/angiotensin-(1-7)/mas receptor Axis in the vascular progenitor cells for cardiovascular diseases. *Molecular Pharmacology*, mol.119.117580. 10.1124/mol.119.117580 PMC7725063 32321734

[16] Swanson KV, Deng M, Ting JP (2019). The NLRP3 inflammasome: Molecular activation and regulation to therapeutics. Nat Rev Immunol.

[17] Ratajczak MZ, Bujko K, Cymer M, Thapa A, Adamiak M, Ratajczak J, Abdel-Latif AK, Kucia M (2020). The Nlrp3 inflammasome as a "rising star" in studies of normal and malignant hematopoiesis. Leukemia.

[18] Franchi L, Munoz-Planillo R, Nunez G (2012). Sensing and reacting to microbes through the inflammasomes. Nat Immunol.

[19] Ratajczak MZ, Adamiak M, Thapa A, Bujko K, Brzezniakiewicz-Janus K, Lenkiewicz AM (2019). NLRP3 inflammasome couples purinergic signaling with activation of the complement cascade for the optimal release of cells from bone marrow. Leukemia.

[20] Lenkiewicz AM, Adamiak M, Thapa A, Bujko K, Pedziwiatr D, Abdel-Latif AK, Kucia M, Ratajczak J, Ratajczak MZ (2019). The Nlrp3 Inflammasome orchestrates mobilization of bone marrow-residing stem cells into peripheral blood. Stem Cell Rev Rep.

[21] Mori J, Oudit GY, Lopaschuk GD (2020). SARS-CoV-2 perturbs the renin-angiotensin system and energy metabolism. American Journal of Physiology. Endocrinology and Metabolism.

[22] Sagulenko V, Thygesen SJ, Sester DP, Idris A, Cridland JA, Vajjhala PR, Roberts TL, Schroder K, Vince JE, Hill JM, Silke J, Stacey KJ (2013). AIM2 and NLRP3 inflammasomes activate both apoptotic and pyroptotic death pathways via ASC. Cell Death Differ.

[23] Ratajczak MZ, Kucia M (2020). SARS-CoV-2 infection and overactivation of Nlrp3 inflammasome as a trigger of cytokine "storm" and risk factor for damage of hematopoietic stem cells. Leukemia.

[24] Ratajczak J, Zuba-Surma E, Klich I, Liu R, Wysoczynski M, Greco N, Kucia M, Laughlin MJ, Ratajczak MZ (2011). Hematopoietic differentiation of umbilical cord blood-derived very small embryonic/epiblast-like stem cells. Leukemia Aug.

[25] Kassmer SH, Jin H, Zhang PX, Bruscia EM, Heydari K, Lee JH, Kim CF, Kassmer SH, Krause DS (2013). Very small embryonic-like stem cells from the murine bone marrow differentiate into epithelial cells of the lung. Stem Cells.

[26] Guerin CL, Loyer X, Vilar J, Cras A, Mirault T, Gaussem P, Silvestre JS, Smadja DM (2015). Bone-marrow-derived very small embryonic-like stem cells in patients with critical leg ischaemia: Evidence of vasculogenic potential. Thromb Haemost.

[27] Chen ZH, Lv X, Dai H, Liu C, Lou D, Chen R, Zou GM (2015). Hepatic regenerative potential of mouse bone marrow very small embryonic-like stem cells. J Cell Physiol.

[28] Havens AM, Shiozawa Y, Jung Y, Sun H, Wang J, McGee S, Mishra A, Taichman LS, Danciu T, Jiang Y, Yavanian G, Leary E, Krebsbach PH, Rodgerson D, Taichman RS (2013). Human very small embryonic-like cells generate skeletal structures, in vivo. Stem Cells Dev.

[29] Wojakowski W, Tendera M, Kucia M, Zuba-Surma E, Paczkowska E, Ciosek J, Hałasa M, Król M, Kazmierski M, Buszman P, Ochała A, Ratajczak J, Machaliński B, Ratajczak MZ (2009). Mobilization of bone marrow-derived Oct-4+ SSEA-4+ very small embryonic-like stem cells in patients with acute myocardial infarction. J Am Coll Cardiol.

[30] Kuruca SE, Celik DD, Ozerkan D, Erdemir G (2019). Characterization and isolation of very small embryonic-like (VSEL) stem cells obtained from various human hematopoietic cell sources. Stem Cell Rev Rep.

[31] Chang YJ, Tien KE, Wen CH, Hsieh TB, Hwang SM (2014). Recovery of CD45(−)/Lin(−)/SSEA-4(+) very small embryonic-like stem cells by cord blood bank standard operating procedures. Cytotherapy.

[32] Monti M, Imberti B, Bianchi N, Pezzotta A, Morigi M, Del Fante C (2017). A novel method for isolation of pluripotent stem cells from human umbilical cord blood. Stem Cells Dev.

[33] Ganguly R, Metkari S, Bhartiya D (2018). Dynamics of bone marrow VSELs and HSCs in response to treatment with gonadotropin and steroid hormones, during pregnancy and evidence to support their asymmetric/symmetric cell divisions. Stem Cell Rev Rep.

[34] Shaikh A, Nagvenkar P, Pethe P, Hinduja I, Bhartiya D (2015). Molecular and phenotypic characterization of CD133 and SSEA4 enriched very small embryonic-like stem cells in human cord blood. Leukemia.

[35] Virant-Klun I (2018). Functional testing of primitive oocyte-like cells developed in ovarian surface epithelium cell culture from small VSEL-like stem cells: Can they be fertilized one day?. Stem Cell Rev Rep.

[36] Park C, Ma YD, Choi K (2005). Evidence for the hemangioblast. Exp Hematol.

[37] Lis R, Karrasch CC, Poulos MG, Kunar B, Redmond D, Duran JGB (2017). Conversion of adult endothelium to immunocompetent haematopoietic stem cells. Nature.

[38] Ribatti D (2008). Hemangioblast does exist. Leuk Res.

[39] Lacaud G, Kouskoff V (2017). Hemangioblast, hemogenic endothelium, and primitive versus definitive hematopoiesis. Exp Hematol.

[40] Guthrie SM, Curtis LM, Mames RN, Simon GG, Grant MB, Scott EW (2005). The nitric oxide pathway modulates hemangioblast activity of adult hematopoietic stem cells. Blood.

[41] Zambidis ET, Park TS, Yu W, Tam A, Levine M, Yuan X (2008). Expression of angiotensin-converting enzyme (CD143) identifies and regulates primitive hemangioblasts derived from human pluripotent stem cells. Blood.

[42] Latz E, Xiao TS, Stutz A (2013). Activation and regulation of the inflammasomes. Nat Rev Immunol.

[43] Groslambert M, Py BF (2018). Spotlight on the NLRP3 inflammasome pathway. Journal of Inflammation Research.

[44] Jo EK, Kim JK, Shin DM, Sasakawa C (2016). Molecular mechanisms regulating NLRP3 inflammasome activation. Cell Mol Immunol.

[45] Suresh R, Chandrasekaran P, Sutterwala FS, Mosser DM (2016). Complement-mediated 'bystander' damage initiates host NLRP3 inflammasome activation. J Cell Sci.

[46] Roh JS, Sohn DH (2018). Damage-associated molecular patterns in inflammatory diseases. Immune Netw.

[47] Gong T, Liu L, Jiang W, Zhou R (2020). DAMP-sensing receptors in sterile inflammation and inflammatory diseases. Nat Rev Immunol.

[48] Meng Y, Pan M, Zheng B, Chen Y, Li W, Yang Q, Zheng Z, Sun N, Zhang Y, Li X (2019). Autophagy attenuates angiotensin II-induced pulmonary fibrosis by inhibiting redox imbalance-mediated NOD-like receptor family Pyrin domain containing 3 Inflammasome activation. Antioxid Redox Signal.

[49] Lim S, Lee ME, Jeong J, Lee J, Cho S, Seo M, Park S (2018). sRAGE attenuates angiotensin II-induced cardiomyocyte hypertrophy by inhibiting RAGE-NFkappaB-NLRP3 activation. Inflamm Res.

[50] Kohlstedt K, Gershome C, Friedrich M, Muller-Esterl W, Alhenc-Gelas F, Busse R, Fleming I (2006). Angiotensin-converting enzyme (ACE) dimerization is the initial step in the ACE inhibitor-induced ACE signaling cascade in endothelial cells. Mol Pharmacol.

[51] Slukvin II (2009). Renin-angiotensin system and hemangioblast development from human embryonic stem cells. Expert Rev Hematol.

[52] Jokubaitis VJ, Sinka L, Driessen R, Whitty G, Haylock DN, Bertoncello I (2008). Angiotensin-converting enzyme (CD143) marks hematopoietic stem cells in human embryonic, fetal, and adult hematopoietic tissues. Blood.

[53] Mizuochi C, Fraser ST, Biasch K, Horio Y, Kikushige Y, Tani K, Akashi K, Tavian M, Sugiyama D (2012). Intra-aortic clusters undergo endothelial to hematopoietic phenotypic transition during early embryogenesis. PLoS One.

[54] Ratajczak, M. Z., Ratajczak, J., & Kucia, M. (2019). Very small embryonic-like stem cells (VSELs) an update and future directions. *Circulation Research, 124*(2), 208–210. 10.1161/CIRCRESAHA.118.314287 PMC6461217 30653438

